# The Effects of Prednisolone Treatment on Cytokine Expression in Patients with Erythema Nodosum Leprosum Reactions

**DOI:** 10.3389/fimmu.2018.00189

**Published:** 2018-02-09

**Authors:** Edessa Negera, Stephen L. Walker, Kidist Bobosha, Yonas Bekele, Birtukan Endale, Azeb Tarekegn, Markos Abebe, Abraham Aseffa, Hazel M. Dockrell, Diana N. Lockwood

**Affiliations:** ^1^Faculty of Infectious Tropical Diseases, London School of Hygiene and Tropical Medicine, London, United Kingdom; ^2^Armauer Hansen Research Institute, Addis Ababa, Ethiopia

**Keywords:** cytokines, erythema nodosum leprosum, leprosy, prednisolone, reaction, treatment, type-2 reaction

## Abstract

Erythema nodosum leprosum (ENL) is a systemic inflammatory complication occurring mainly in patients with lepromatous leprosy (LL) and borderline lepromatous leprosy. Prednisolone is widely used for treatment of ENL reactions but clinical improvement varies. However, there is little good *in vivo* data as to the effect of prednisolone treatment on the pro-inflammatory cytokines in patients with ENL reactions. As a result, treatment and management of reactional and post-reactional episodes of ENL often pose a therapeutic challenge. We investigated the effect of prednisolone treatment on the inflammatory cytokines TNF, IFN-γ, IL-1β, IL-6, and IL-17 and the regulatory cytokines IL-10 and TGF-β in the skin lesion and blood of patients with ENL and compared with non-reactional LL patient controls. A case–control study was employed to recruit 30 patients with ENL and 30 non-reactional LL patient controls at ALERT Hospital, Ethiopia. Blood and skin biopsy samples were obtained from each patient before and after prednisolone treatment. Peripheral blood mononuclear cells from patients with ENL cases and LL controls were cultured with *M. leprae whole-cell sonicates* (MLWCS), phytohemagglutinin or no stimulation for 6 days. The supernatants were assessed with the enzyme-linked immunosorbent assay for inflammatory and regulatory cytokines. For cytokine gene expression, mRNA was isolated from whole blood and skin lesions and then reverse transcribed into cDNA. The mRNA gene expression was quantified on a Light Cycler using real-time PCR assays specific to TNF, IFN-γ, IL-β, TGF-β, IL-17A, IL-6, IL-8, and IL-10. The *ex vivo* production of the cytokines: TNF, IFN-γ, IL-1β, and IL-17A was significantly increased in untreated patients with ENL. However, IL-10 production was significantly lower in untreated patients with ENL and significantly increased after treatment. The *ex vivo* production of IL-6 and IL-8 in patients with ENL did not show statistically significant differences before and after prednisolone treatment. The mRNA expression in blood and skin lesion for TNF, IFN-γ, IL-1β, IL-6, and IL-17A significantly reduced in patients with ENL after treatment, while mRNA expression for IL-10 and TGF-β was significantly increased both in blood and skin lesion after treatment. This is the first study examining the effect of prednisolone on the kinetics of inflammatory and regulatory cytokines in patients with ENL reactions before and after prednisolone treatment. Our findings suggest that prednisolone modulates the pro-inflammatory cytokines studied here either directly or through suppression of the immune cells producing these inflammatory cytokines.

## Introduction

Leprosy is a chronic disease caused by *Mycobacterium leprae*, an acid-fast bacillus whose clinical spectrum correlates with the host immune response. It mainly infects the skin and peripheral nerves. There are five-clinical forms of leprosy called spectrum with the localized tuberculoid leprosy (TT) and the generalized lepromatous leprosy (LL) forming the two poles of the spectrum (1). Erythema nodosum leprosum (ENL) is an immune-mediated inflammatory complication causing high morbidity in affected leprosy patients (2). The clinical manifestation of ENL includes tender erythematous crops of skin lesions and systemic features of disease including fever, neuritis and bone pain (3). ENL occurs mainly in patients with LL and borderline lepromatous (BL) leprosy before, during, or after successful completion of multidrug treatment (MDT) (4).

Erythema nodosum leprosum reaction is associated reportedly with changes in cytokine profiles (5, 6). Cytokines are low molecular weight soluble proteins which mediate the cross-talk between the different cells of the immune system. They play a central role in the recruitment of the immune cells, the clonal development of the lymphocytes, the innate immune response and the effector response of most immune cells. These complex regulatory networks of cytokines often determine the clinical course of infections and the outcome (7).

In leprosy, cytokine research focused mainly on the association of different cytokine profile with the spectrum of the disease specifically with the Th1–Th2 cytokine profiles (5, 8, 9). The immune response to *M. leprae* in tuberculoid (TT) patients is associated with Th1 cytokines (IFN-γ and TNF) whereas LL patients are characterized by Th2 (IL-4, IL-5) cytokines production (6, 10–15). However, recent studies have shown that a distinct cytokine profile associated with a specific clinical form and reactions has not been attained (16–18).

The immune response to *M. leprae* is cytokine mediated, whereas the involvement of cytokines in ENL reaction is less understood. Literature reveals controversial results on the cytokines profile which are involved in ENL reaction. The presence of both Th1- and Th2-type cytokines in ENL lesions as well as in the sera of ENL patients has been reported by some authors (19–21). However, others reported that only Th1 cytokines are involved in the pathogenesis of ENL (6, 19, 22, 23). Yet other authors suggested that there is no clear association of either Th1 or Th2 cytokine secretion profile in leprosy patients with ENL reactions compared to non-reactional LL patients (8). In addition to the Th1- and Th2-driven cytokines, IL-17 has been identified as a new subset of cytokine recently reported associated with ENL reactions (24–26).

Erythema nodosum leprosum reaction is treated with prednisolone or with thalidomide. Thalidomide is not available in most leprosy endemic countries such as Ethiopia due to its severe side effects. Prednisolone is an immunosuppressor drug used for the treatment of chronic inflammatory diseases. The anti-inflammatory role of prednisolone is mainly due to its ability to suppress or inhibit the activation of transcription factors NF-kβ (27). NF-kβ regulates genes encoding for IL-1β, TNF, IL-2, and inducible nitric oxide synthase (28, 29). In leprosy reactions, although the results are conflicting, treatment of ENL with prednisolone has been correlated with downregulation of inflammatory cytokines, such as IL-1β, TNF, IFN-γ, and IL-17 (20, 22, 30). On the other hand, other studies reported the selective downregulation of regulatory cytokines after prednisolone treatment (6, 31).

Although oral prednisolone is widely used for treatment of ENL, clinical improvement varies in which more than 40% of cases do not show clinical improvement (32). High recurrent episodes and flare-ups are common in these patients. However, there is little good *in vivo* data as to the effect of prednisolone treatment on the pro-inflammatory cytokines. As a result, treatment and management of reactional and post-reactional episodes of ENL often pose a therapeutic challenge to leprologists.

We investigated the effect of prednisolone treatment on the inflammatory cytokines TNF, IFN-γ, IL-1β, IL-6, and IL-17 and the regulatory cytokines IL-10 and TGF-β in the skin lesion and blood of patients with ENL and compared with non-reactional LL patient controls. Our aim was to compare events at the local site of infection (i.e., the skin lesion) and the systemic effects of the prednisolone on the cytokine production and their gene expression before, during, and after treatment. LL patients without reactions were included as controls. We hypothesized that prednisolone treatment would downregulate the inflammatory cytokines and upregulate regulatory cytokines both locally in the skin and systemically in the blood and hence establish cytokine homeostasis.

## Materials and Methods

### Study Design

A case–control study with follow-up for 28 weeks after the initiation of prednisolone treatment was used to recruit 30 patients with ENL reaction and 30 non-reactional LL patient controls between December 2014 and January 2016 at ALERT hospital, Ethiopia.

### Ethical Considerations

Informed written consent for blood and skin lesion biopsies were obtained from patients following approval of the study by the Institutional Ethical Committee of London School of Hygiene and Tropical Medicine, UK (#6391), AHRI/ALERT Ethics Review Committee, Ethiopia (P032/12) and the National Research Ethics Review Committee, Ethiopia (#310/450/06). All patient data were analyzed and reported anonymously.

### Patient Recruitment

Leprosy patients were recruited at ALERT Hospital, Addis Ababa, Ethiopia. The patients were classified clinically and histologically on the leprosy spectrum based on the Ridley–Jopling (RJ) classification schemes (1). ENL was clinically diagnosed when a patient with BL or LL leprosy had painful crops of tender cutaneous erythematous skin lesions (3). New ENL was defined as the occurrence of ENL for the first time in a patient with LL or BL. LL was clinically diagnosed when a patient had widely disseminated nodular lesions with ill-defined borders and BI above 2 (2). Patients with ENL were treated according to the World Health Organization (WHO) treatment guideline with steroids that initially consisted of 40 mg oral prednisolone daily and the dose was tapered by 5 mg every fortnight for 24 weeks. All patients were received WHO-recommended leprosy MDT.

### Blood and Skin Lesion Biopsy Samples

Twenty microliters of venous blood were collected into sterile BD Heparinized Vacutainer^®^ tubes (BD, Franklin, Lakes, NJ, USA) before treatment, during treatment on week 12, and after treatment on week 24 from each patient and used for peripheral blood mononuclear cell (PBMC) isolation. In addition, 2 mL of blood was collected into PAXgene^®^ Blood RNA Tubes (PreAnalytix, GmbH, Switzerland) before, during, and after prednisolone treatment for mRNA isolation and stored at −80°C. Six-millimeter punch biopsy was taken from each patient before and after prednisolone treatment into a Nunc^®^ tube containing 1 mL RNAlater^®^ solution (Thermo-Fisher Scientific) and was kept at −20°C for 48 h and then transferred to −80°C freezer. ENL and LL lesions for biopsy sample were identified and marked by a dermatologist and then biopsy samples were taken from the marked area by trained research nurses under supervision.

### PBMC Isolation, Storage, and Thawing

Peripheral blood mononuclear cells were separated by density gradient centrifugation at 800 × *g* for 25 min on Ficoll–Hypaque (Histopaque, Sigma Aldrich, UK) as described earlier (33, 34). Cells were washed three times in sterile 1× phosphate-buffered saline (Sigma Aldrich^®^, UK) and re-suspended with 1 mL of Roswell Park Memorial Institute (RPMI medium 1640 (1×) + GlutaMAX™ + Pen-Strip GBICO™, Life technologies™, UK). Cell viability was determined by 0.4% sterile Trypan Blue solution (Sigma Aldrich^®^, UK), the viability was between 94 and 98%. PBMC freezing was performed using a cold freshly prepared freezing medium composed of 20% fetal bovine serum (FBS, heat inactivated, endotoxin tested ≤5 EU/ml, GIBCO^®^ Life technologies, UK), 20% dimethyl sulfoxide in RPMI medium 1640 (1×). Cells were kept at −80°C for 2–3 days and transferred to liquid nitrogen until use. Cell thawing was done as described (35). The procedure is briefly described as follows: cells were incubated in a water bath (37°C) for 30 s until thawed half way and re-suspended in 10% FBS in RPMI medium 1640 (1×) (37°C) containing 1/10,000 benzonase until completely thawed, washed two times (5 min each), and counted. The percentage viability obtained was above 90%.

### PBMC Stimulation with *M. leprae* Whole-Cell Sonicate (WCS)

Total PBMCs (200,000 cells/well) were added in triplicate into 96-well U-bottom tissue culture plates and cultured with 10 mg/mL irradiated armadillo-derived *M. leprae* WCS (kindly supplied by Dr. J. S. Spencer through the NIH/NIAID “Leprosy Research Support” Contract N01 AI-25469 from Colorado State University), 1 mg/mL phytohemagglutinin or AIM-V medium at 37°C with 5% CO_2_ and 70% humidity. After 6 days, supernatants were collected and kept frozen until used in enzyme-linked immunosorbent assay (ELISA).

### Cytokine Measurement by ELISA

Supernatants were tested for cytokines using a Ready-Set-Go! ^®^ Sandwich ELISA. Capture and biotinylated detection antibodies directed against IFN-γ, TNF, IL-1β, IL-6, IL-10, IL-10, and IL-17A were purchased from eBioscience (Affymetrix, eBioscience, UK). A 96-well flat-bottom Nunc MaxiSorp^®^ ELISA plates (Affymetrix, eBioscience, UK) were used. Standards for each cytokines were prepared by serial dilution as recommended by the supplier (Affymetrix, eBioscience, UK). Detection was performed with avidin-horseradish peroxidase (Avidin-HRP) conjugated with tetramethylbenzidine following the supplier’s procedure (Affymetrix, eBioscience, UK). For all plates, the optical density (OD) at 450 nm was measured using an ELISA plate reader (Microplate reader; Bio-Rad, Richmond, CA, USA). A curve fit was applied to each standard curve according to the manufacturer’s manual. Sample concentrations were interpolated from these standard curves. The assays were sensitive to over concentration ranges from 2 to 200 pg/mL for IL-6 and IL-17A, from 2 to 250 pg/mL for IL-8, from 2 to 300 pg/mL for IL-10, and from 4 to 500 pg/mL for TNF, IFN-γ, and IL-1β.

### RNA Isolation and Reverse Transcription

Isolation of RNA from whole blood and skin lesion biopsies stored in RNAlater™ (Ambion, Austin, TX, USA) was performed using PAXgene Blood RNA Kit and RNeasy Fibrous Tissue Kit (QIAGEN Crawley, West Sussex, United Kingdom), respectively, according to the manufacturer’s protocol. DNase I (QIAGEN) was included for all RNA preparations for DNA digestion. RNA yield was determined using a NanoDrop 2000, spectrophotometer (Thermo Scientific, Epsom, UK) and integrity was checked by agarose gel electrophoresis. For all samples, complementary DNA (cDNA was synthesized on the same day to avoid the risk of RNA degrades during storage). cDNA was synthesized from RNA (200 ng/reaction mixture) using High Capacity cDNA Reverse Transcriptase Kit (AB Applied Biosystems, UK). Reactions were incubated in an ABI9700 Programmable Thermal Cycler (Applied Biosystems, Foster City, CA, USA) for 10 min at 25°C followed by 120 min at 37°C and 5 min at 85°C and then cooling to 4°C.

### Primers and Quantitative Polymerase Chain Reaction

Primers between 20 and 24 nt in length were designed across intron/exon boundaries on mRNA sequence obtained from the Nation Centre for Biotechnology Information database (NCBI) to give a product between 100–500 bp. All primer sequences were blasted on the NCBI data bank to confirm their specificity. Custom synthesis of oligonucleotide primers was performed by Sigma-life science and provided in desalted form. The nucleotide sequences of the forward and reverse primers, respectively, used in this study were as follows: for IL-10, 5′-TGAGAACCAAGACCCAGACA-3′ and 5′-TCATGGCTTTGTAGATGCCT-3′; for TNF, 5′-AGCCCATGTTGTAGCAAACC-3′ and 5′-GCTGGTTATCTCTCAGCTCCA-3′; for IL-17A, 5′-AGACCTCATTGGTGTCACTGC-3′ and 5′-CTCTCAGGGTCCTCATTG CG-3′; for Il-6, 5′-TTCGGTCCAGTTGCCTTCTC-3′ and 5′-TACATGTCTCCTTTCTCA GGGC-3′; or IL-1β, 5′-AGCCCCAGCCAACTCAATTC-3′ and 5′-CATGGAGAACAC CACTTGTTGC-3′; for IFN-γ: 5′-GGCTTTTCAGCTCTGCATCG-3′ and 5′-TCTGTCAC TCTCCTCTTTCCA-3′; for IL-8: 5′-ACCGGAAGGAACCATCTCAC-3′ and 5′- AAAC TGCACCTTCACACAGAG-3′; for TGF-β: 5′-ACATCAACGCAGGGTTCACT-3′ and 5′- GAAGTTGGCATGGTAGCCC-3′; for human acidic ribosomal protein (HuPO) housekeeping gene: 5′-GGACTCGTTTGTACCCGTTG-3′ and 5′-GGACTCGTTTGTA CC CG TTG-3′.

**Figure 1:**
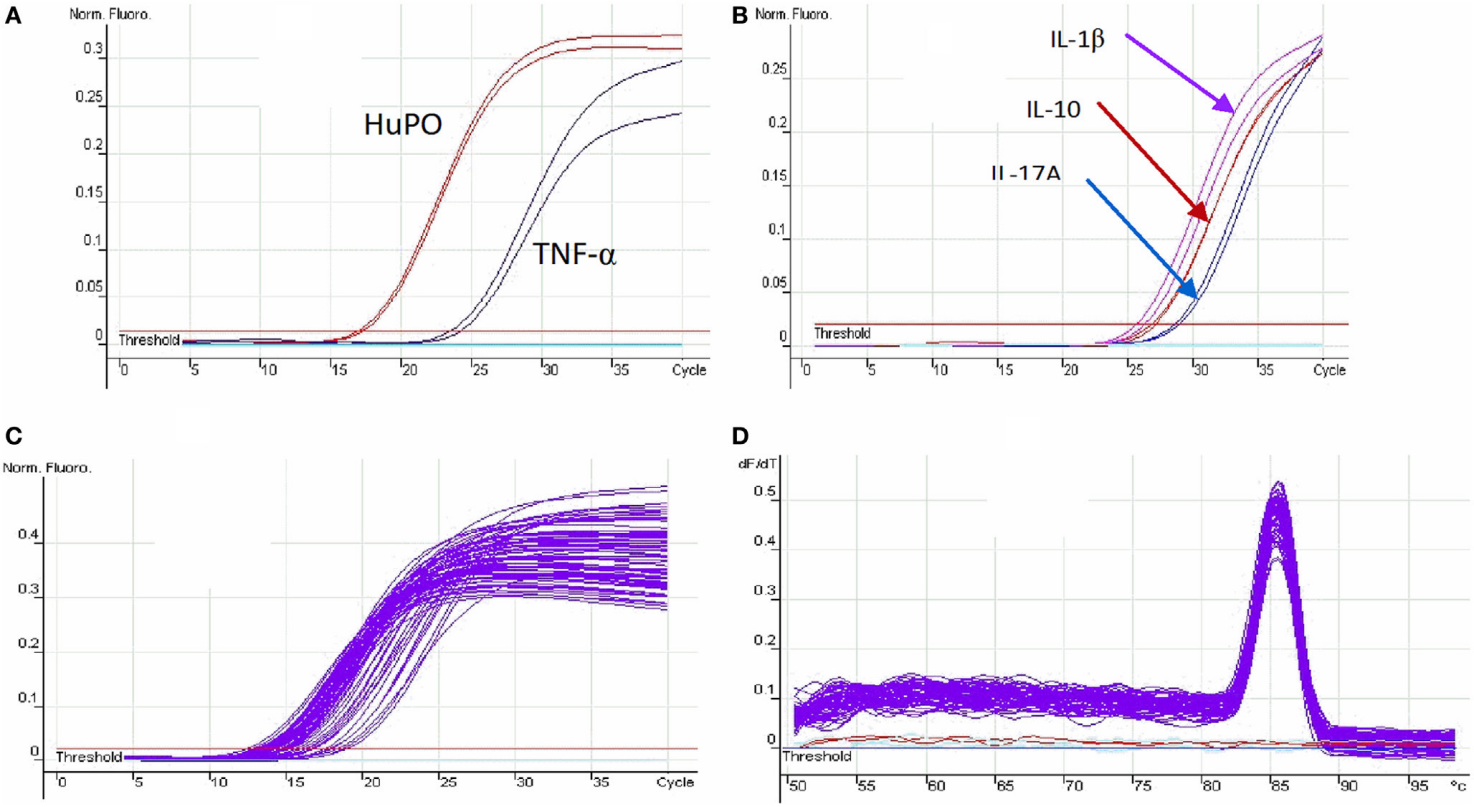
PCR optimization: **(A)** the optimization threshold of housekeeping gene (HuPO) and TNF-α; **(B)** the optimization cycle threshold for IL-1β, IL-10, and IL-17A; **(C)** the cycle threshold of housekeeping gene for multiple samples; and **(D)** the melt curve analysis. The peak curve indicates the primer amplified the same region of all samples and primer-dimer is not detected as there is only one such peak.

Real-time quantitative PCR for all genes was performed on the Rotor-Gene™ 3000 programmable thermal cycler (Corbett Life Science, Qiagen, Crawley, UK) using Roter-gene^®^ SYBR^®^ Green PCR Kit (Qiagen, Crawley, UK). The Rotor-Gene conditions were set as follows: Initial activation step (polymerase activation) was achieved by incubating at 95°C for 15 min, 40 cycles of denaturation at 95°C for 5 s, annealing at 60°C for 10 s, extension at 72°C for 20 s, and fluorescence acquisition for 5 s at 72°C. The primer-dimer formation was checked by melting curve analysis. Melting point data were obtained by increasing the temperature from 50°C to 99°C by 1°C on each step. The interval between increases in temperature was 30 s for the first step and then 5 s for subsequent steps. An assay control was included from mRNA extraction to the amplification steps. For mRNA extraction, one assay control per batch was used. The assay control included all buffers except the sample and was processed under identical conditions with the samples. The same assay control was used during cDNA synthesis and real-time quantitative PCR (Figure 1).

### Statistical Methods

#### ELISA Data

The OD of each sample for each cytokine was obtained by the ELISA reader. The OD was converted to concentration (pg/mL) by microplate manager 6. Unpaired *t*-test was used to compare the relative concentration of the cytokines production in patients with ENL and LL controls. For comparing the cytokine concentration in patients with ENL before and after treatment, paired t-test was used. Results are presented as mean ± SEM with *P*-values with a cut–off 0.05. SE was chosen since the primary objective of the study was to measure how the mean of the sample is related to the mean of the underlying population. SE takes SD and sample size into account.

#### Real-time Quantitative PCR

Real-time quantitative PCR for the mRNA gene expression of target genes, the relative threshold cycle value (C_T_) comparison method was used. The relative gene expression was analyzed by using the 2^−ΔΔ CT^ method (36). The C_T_ value is the threshold number for the amplification of the target gene. Cikos et al. compared the six different methods currently used for real-time PCR data analysis and has shown that the best results were obtained with the relative standard curve (ΔC_T_) method and the least coefficient of variation (ΔCV). The C_T_ values were obtained for the target gene and control gene (HuPO) for each patient sample at each time point. Then, the difference in C_T_ value was obtained by subtracting the C_T_ of the target gene from the C_T_ of the control gene and designated as ΔC_T_. To compare the target gene expression in patients with ENL and LL controls, ΔΔC_T_ was obtained by subtracting the ΔC_T_ of LL patient control from the ΔC_T_ of the patient with ENL. Then, the fold change was obtained by using the formula 2^−ΔΔ CT^. similarly, for the comparison of the relative target gene expression in patients with ENL before and after treatment, ΔΔC_T_ was obtained by ΔC_T_ (after) minus ΔC_T_ (before). Then the fold change for target gene expression from the baseline (before treatment) was given by 2^−ΔΔ CT^. Unpaired *t*-test was used to compare the fold change of each target gene for patients with ENL compared to LL patient controls. To compare the expression level of the desired gene in patients with ENL before and after treatment, paired *t*-test was used.

## Results

Results are presented in two sections. First, we presented the results of cytokine production and then followed by the gene expressions. Within each section, we presented first the comparisons made between ENL and LL controls and then followed by the comparisons made within ENL patients at different time points.

### Patient Clinical Background

Thirty LL patients with ENL reaction and 30 LL patient controls without ENL reaction were recruited between December 2014 and January 2016. The male to female ratio was 2:1 with a median age of 27.5 (range: 18–56) years in patients with ENL and 3:1 with a median age of 25.0 (range: 18–60) years in patients with non-reactional LL controls. All ENL patients were untreated with corticosteroid before recruitment. At time of recruitment, 10 ENL patients were previously untreated with MDT, 15 are on MDT and 5 were completed MDT treatment. Twenty-one LL patients were about to start MDT, 9 were on MDT at recruitment.

### Increased *In Vitro* Inflammatory Cytokine Production in Untreated ENL Patients Compared to LL Controls

**Figure 2:**
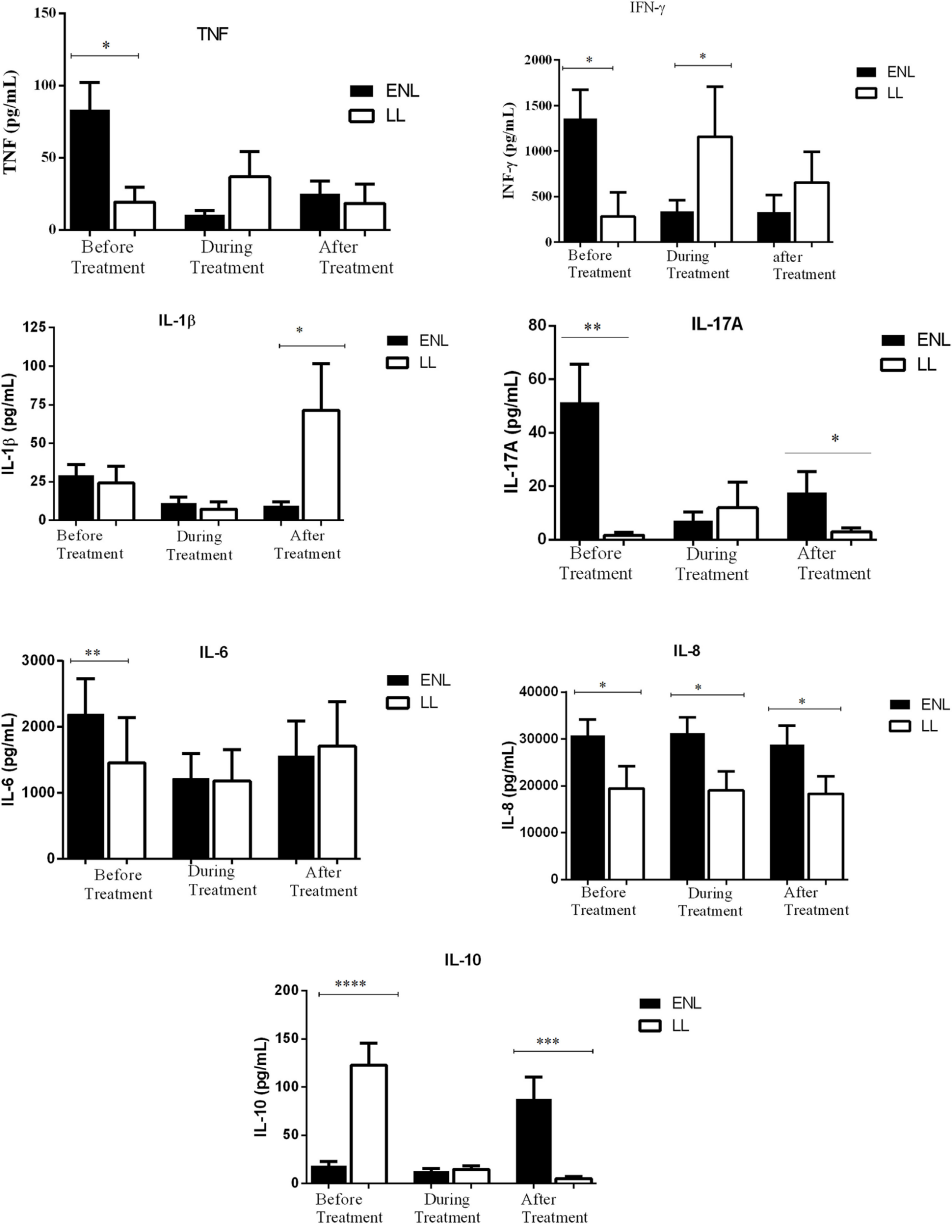
Comparison of the levels of *in vitro* production of TNF, IFN-γ, IL-β and IL-17A, IL-6, IL-8, and Ll-10 in culture supernatants of peripheral blood mononuclear cells from patients with erythema nodosum leprosum (ENL) and lepromatous leprosy (LL) controls before and after treatment. Statistical test: unpaired *t*-test, α = 0.05. *P ≤ *0.05*; ** *P* ≤ *0.005*. Bar graphs show mean ± SEM.

The mean production of TNF in response to *M. leprae* WCS stimulation was significantly higher (83.6.4 ± 18.82 pg/mL) in the culture supernatants of PBMCs from untreated ENL patients than from LL patient controls (19.4 pg/mL ± 10.44) (*P* ≤ 0.05). During treatment, the level of TNF production decreased to 10.7 pg/mL in patients with ENL while it was increased to 36. 9 pg/mL in LL patient controls. After treatment, TNF production was not significantly different between the two groups (Figure 2).

Patients with ENL reactions had a significantly higher (1361 pg/mL ± 309.6) IFN-γ production than the LL patient controls (280.1 pg/mL ± 269.80) before treatment (*P* ≤ 0.05). However, during treatment, the level of IFN-γ production was significantly decreased in patients with ENL to 304.4 pg/mL ± 119.6 while it was increased in LL patient controls to 1158.0 pg/mL ± 549.2 (*P* ≤ 0.05). The level of IFN-γ production was not significantly different in both groups after treatment (Figure 2).

Although the amount of IL-1β production was similar in patients with ENL and LL controls before and during treatment, patients with ENL had much lower production of IL-1β than LL patient controls after treatment (*P* ≤ 0.05). Higher production of IL-17A was obtained in patients with ENL cases compared to LL patient controls before treatment (*P* ≤ 0.005). Although the *in vitro* production of IL-17A was considerably decreased after treatment in patients with ENL, it still remained higher than in LL patient controls (*P* ≤ 0.05) (Figure 2).

The *in vitro* response of IL-6 production was higher in patients with ENL than in LL controls before treatment (*P* ≤ 0.005). However, during and after treatment, the *in vitro* IL-6 production was not significantly different in both groups. On the other hand, the level of IL-8 production was found to be higher in patients with ENL than in LL controls throughout the study period (*P* ≤ 0.05) (Figure 2).

The IL-10 production was considerably lower in patients with ENL (18.59 pg/mL ± 4.05) than in LL patient controls (122.6 pg/mL ± 23.18) before treatment (*P* < 0.0001). Interestingly, after treatment, the level of IL-10 production was significantly increased to 87.78 pg/mL ± 22.49 in patients with ENL while it was substantially decreased to 4.91 pg/mL ± 2.160 in LL patient controls and the difference was statistically significant (*P* ≤ 0.001) (Figure 2).

In conclusion, the *in vitro* production of the cytokines TNF, IFN-γ, IL-17A, IL-6, and IL-8 were higher in patients with ENL than in LL patient controls before treatment while IL-10 was significantly lower in patients with ENL than in LL patient controls at recruitment.

**Figure 3:**
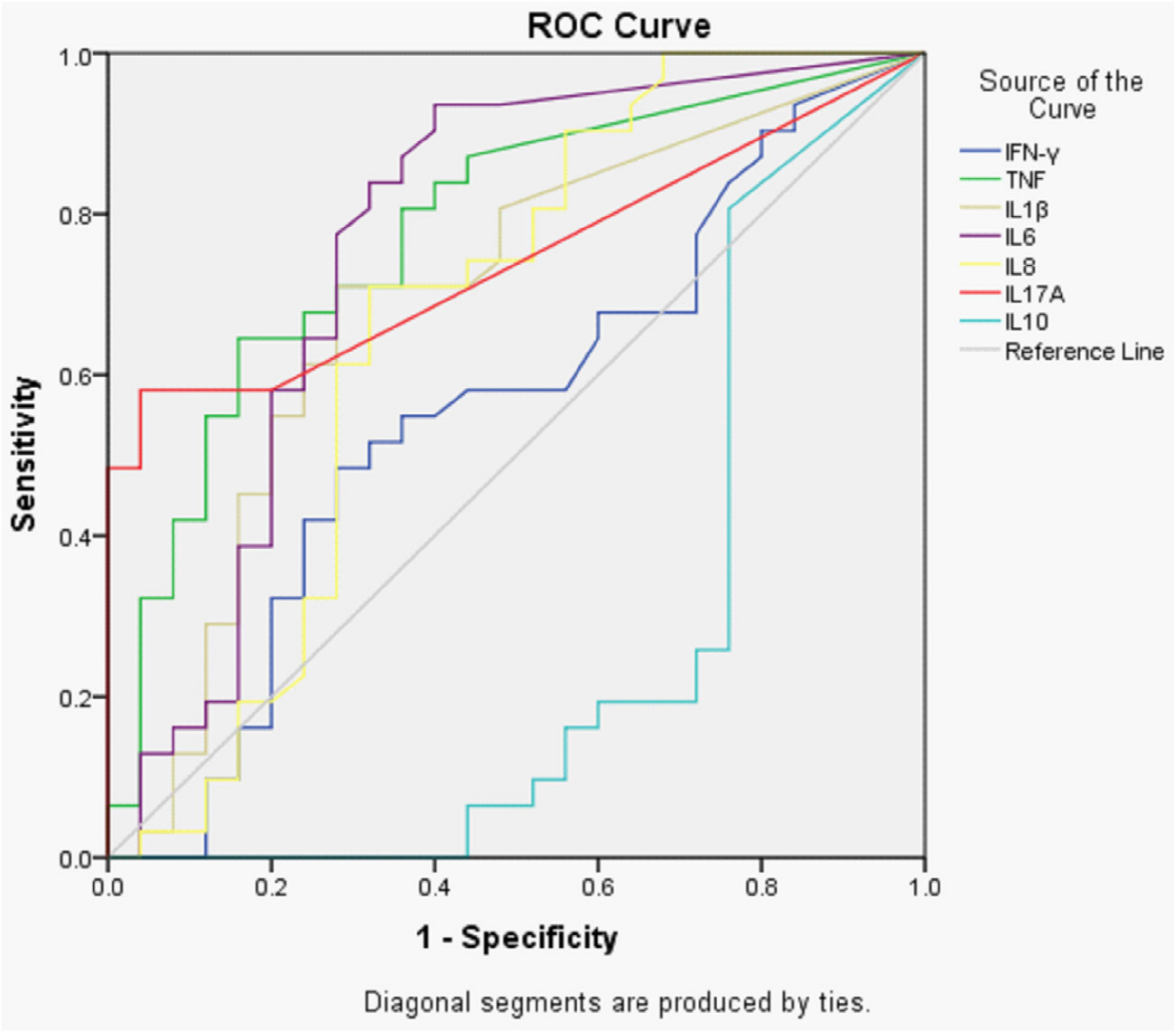
Receiver operator characteristic (ROC) curves showing the accuracies of individual cytokines in discriminating between patients with ENL and LL controls before treatment. AUC = area under the curve.

A receiver operator characteristic (ROC) curve plot for the accuracy of a single cytokine to discriminate between patients with ENL and LL controls was produced. The most accurate cytokines that differentiate between patients with ENL and LL controls were as follows: TNF, IL-6, IL-17A, IL-1β, IL-8, IFN-γ, and IL-10 with the corresponding of area under the curves (AUCs) of 0.779, 0.763, 0.745, 0.695, 0.667, 0.557, and 0.710, respectively (Figure 3).

#### Principal Component Analysis

**Figure 4:**
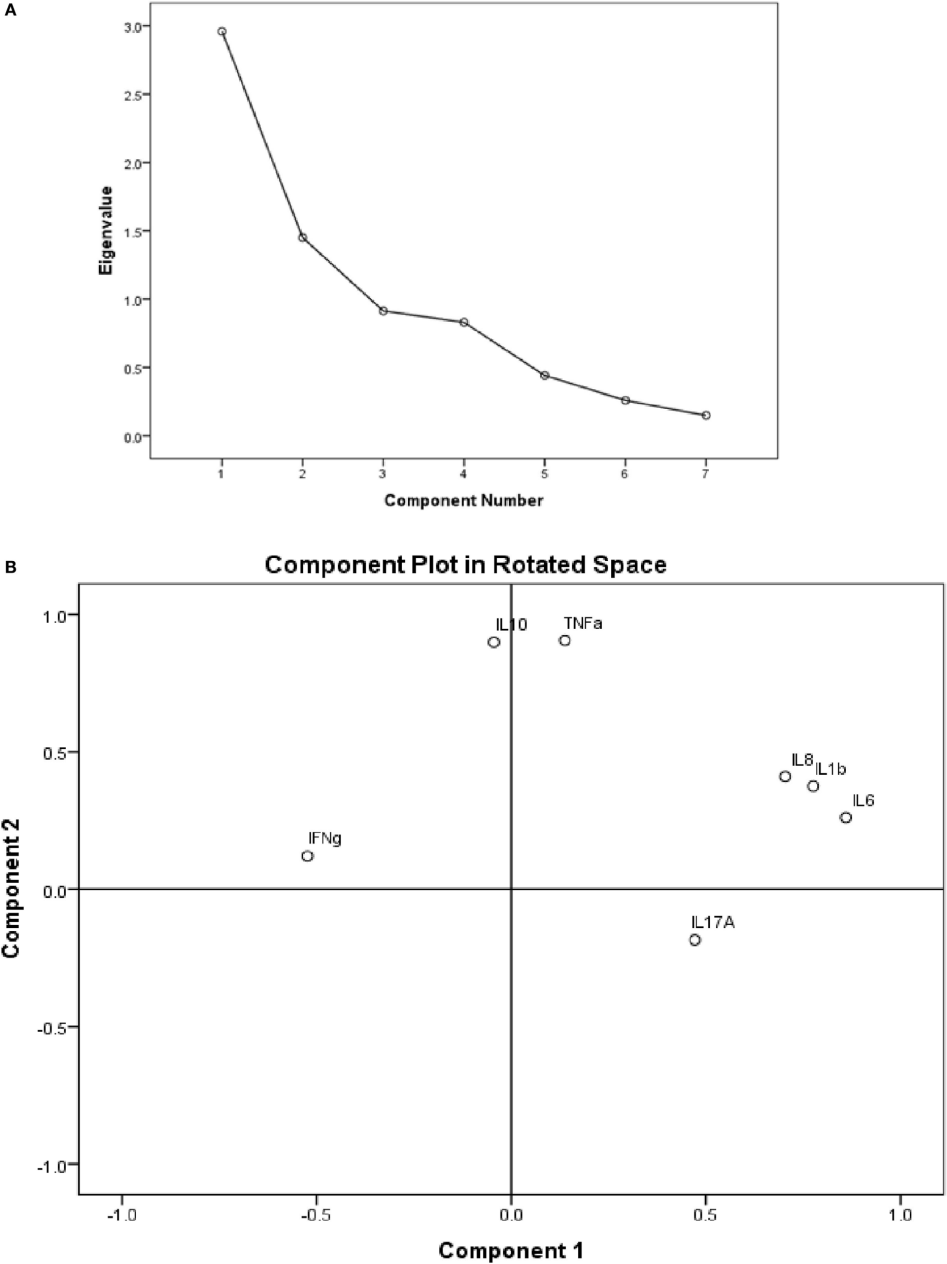
**(A)** Scree plot showing the *in vitro* cytokine production in culture supernatants of peripheral blood mononuclear cells (PBMCs) from patients with erythema nodosum leprosum (ENL). The PCs have eigen values >1 are taken as the most significant components explaining variability in the data; **(B)** the component plot in rotated space showing visual representation of the loadings plotted in a 2-dimensional space.

Principal component analysis with varimax rotation was conducted to assess the cytokine variables in order to summarize them by reducing the dimension of the dataset into principal components (Figure 4A). A loading score statistic was used to select the principal components most associated with the outcome variable (ENL). The assumption of independent sampling was met. The assumptions of normality, linear relationships between pairs of variables, and the variables being correlated at a moderate level were checked.

The component plot in rotated space (Figure 4B) shows how closely related the cytokines are to each other and to the two components. This plot of the component loadings shows that IL-6, IL-1β, and IL-8 all load highly and positively on the first component. IL-10 and TNF had near zero on the first component, but load highly on the second component. IL-17A loads moderately on the first component while IFN-γ loads moderately on component 2. The cytokine levels of IL-6, IL-1β, IL-8, and IL-17 loaded together on the first component with loading coefficient of 0.860, 0.776, 0.704, and 0.472, respectively, explaining 42.274% of the total variation in the cytokine study. Component 2 is determined by three cytokines: IL-10, TNF, and IFN-γ with component loading coefficient of 0.899, 0.906, 0.120, respectively, and explaining 20.714% of the variation.

### Decreased *In Vitro* Inflammatory Cytokine Production in ENL Patients after Prednisolone Treatment

**Figure 5:**
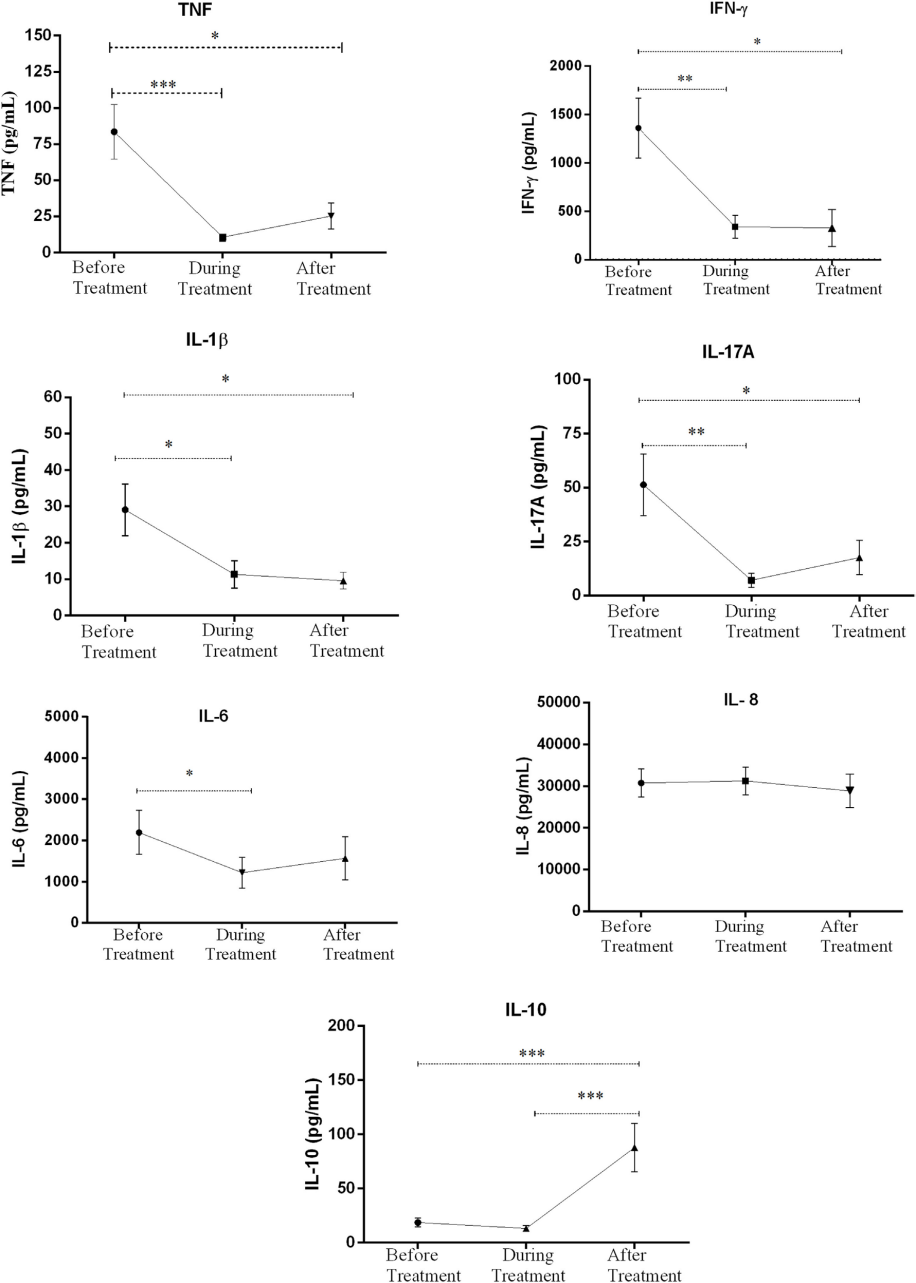
Comparison of the levels of *in vitro* cytokine production in culture supernatants of peripheral blood mononuclear cells (PBMCs) from patients with erythema nodosum leprosum (ENL) before and after treatment. Statistical test: paired *t*-test, α = 0.05. *P ≤ *0.05*; **P ≤ *0.005*; ***P < *0.001*; ****P < *0.0001*. Error bars show mean ± SEM.

The levels of IFN-γ, IL-17A, TNF, IL-6, IL-8, Il-1β, and IL-10 production in response to *M. leprae* WCS before, during, and after treatment were compared within ENL. The *in vitro* TNF production in response to *M. leprae* WCS stimulation was considerably higher (83.6 pg/mL ± 18.82) before treatment than during treatment (10.7 pg/mL ± 2.79) (*P* ≤ 0.001). After treatment, it was slightly increased to 25.4 pg/mL ± 8.88 than during treatment but was still lower than the amount obtained before treatment (*P* ≤ 0.005). Likewise, the *in vitro* production of IFN-γ in response to *M. leprae* WCS was considerably higher before treatment (1361.0 pg/mL ± 309.6) than during treatment (304.4 pg/mL ± 119.6) (*P* ≤ 0.05) and after treatment (328.2 pg/mL ± 190.3) (*P* ≤ 0.05) (Figure 5).

The level of IL-1β and IL-17A production were also found to be higher before treatment than during and after prednisolone treatment of patients with ENL. Although IL-6 production was appreciably decreased during treatment, it was increased after treatment and statistically a significant difference was not revealed before or after treatment. Unlike IL-6, IL-8 production did not show any significant change before, during and after treatment (Figure 5). On the other hand, the level of IL-10 production was very low before and during treatment but substantially increased after treatment (Figure 5).

In summary, the *in vitro* production of IFN-γ, IL-17A, TNF, and IL-1β to *M. leprae* WCS stimulation were higher before prednisolone treatment and have shown a significant reduction after treatment indicating the possible association of these pro-inflammatory cytokines and ENL reaction. On the other hand, the *in vitro* production of IL-10 was noticeably low before treatment and significantly increased after treatment showing its possible regulatory activity. IL-6 and IL-8 did not show significant change before and after treatment of patients with ENL.

### Inflammatory Cytokines Gene Expression Upregulated in the Peripheral Blood of Untreated ENL Patients Compared to LL Controls

To investigate the cytokine gene expression in blood and skin biopsies, a fold change (FC) was used to compare the levels of these gene expressions in patients with ENL and LL controls as well as among patients with ENL before and after treatment.

The gene expression levels of TNF (FC = 3.31), IFN-γ (FC = 2.42), IL-6 (FC = 6.01), IL-8 (FC = 3.19), and IL-17A (FC = 3.58) were significantly increased in the blood samples from patients with ENL compared to LL patient controls before treatment (*P* ≤ 0.05). However, after treatment, the gene expression levels of these cytokines did not reveal statistically a significant difference between ENL patients and LL controls (Table 1).

**Table 1:** Cytokine mRNA expression in the blood samples from patients with erythema nodosum leprosum and lepromatous leprosy controls before and after treatment.

Gene of interest	Before treatment			After treatment		
	ΔΔC_T_	FC	*P*-value	ΔΔC_T_	FC	*P*-value
TNF	−1.73	3.31	0.0047*	0.00	1.00	0.9989
IFN-γ	−1.27	2.42	0.0044*	0.08	0.95	0.8804
IL-1β	−1.23	2.34	0.1085	−0.24	1.18	0.7333
IL-6	−2.59	6.01	0.0003*	−0.99	1.99	0.1167
IL-8	−1.68	3.19	<0.0001*	−0.81	1.75	0.243
IL-10	0.83	0.56	0.1552	0.28	0.82	0.5882
IL-17A	−1.84	3.58	0.0002*	1.12	0.46	0.0502
TGF-β	−0.07	1.05	0.8724	−2.27	4.82	0.0119*

Statistical test: unpaired t-test, α = 0.05, ΔΔC_T_ = delta delta C_T_, FC = fold change, * = significant at α = 0.05.

Although, the fold changes (FC) of mRNA gene expression for IL-1β and TGF-β were slightly increased before treatment in the blood samples from patients with ENL compared to LL patient controls, statistically a significant difference was not revealed. On the other hand, the mRNA gene expression for TGF-β was increased (FC = 4.82) in patients with ENL compared to LL patient controls after treatment (*P* ≤ 0.05). The level of IL-10 gene expression was not significantly different in the two groups before and after treatment (Table 1).

Except IL-10, the result of mRNA gene expression of the other cytokines (TNF, IFN-γ, IL-β, TGF-β, IL-17A, IL-6, and IL-8) was found to be consistent with the result of the corresponding *in vitro* cytokine production. Unlike the IL-10 cytokine production in the culture supernatants in response to *M. leprae* WCS, its gene expression in patients with ENL did not reveal statistically significant difference compared to LL patient controls.

### Comparison of Cytokine Gene Expression in Skin Biopsy Samples from Patients with ENL and LL Controls

The expression of mRNA in skin biopsies for TNF (FC = 2.04), IFN-γ (FC = 4.01), IL-1β (FC = 6.35), IL-6 (FC = 5.30), and IL-17A (FC = 2.99) were significantly higher (*P* ≤ 0.005) in the biopsies from patients with ENL than in the biopsies from LL patient controls before treatment. However, except IL-6 (FC = 2.07, *P* ≤ 0.05), statistically, a significant difference was not obtained in mRNA gene expression for these cytokines in patients with ENL and LL controls after treatment. The mRNA expression for IL-10 was significantly lower (FC = 0.38) in patients with ENL than in LL patient controls (*P* ≤ 0.0001) before treatment but significantly increased after treatment in patients with ENL (FC = 3.5) compared to LL patient controls and the difference was statistically significant (*P* ≤ 0.0001). The mRNA expression for TGF-β and IL-8 in patients with ENL and LL controls did not show statistically significant difference after treatment (Table 2).

**Table 2:** Cytokine mRNA expression in the skin biopsy samples from patients with erythema nodosum leprosum and lepromatous leprosy controls before and after treatment.

Gene of interest	Before treatment			After treatment		
	ΔΔC_T_	FC	*P*-value	ΔΔC_T_	FC	*P*-value
TNF	−1.03	2.04	0.0033*	−0.09	1.07	0.89
IFN-γ	−2.01	4.01	0.0058*	−0.32	1.25	0.5224
IL-1β	−2.67	6.35	0.0006*	−0.24	1.18	0.7333
IL-6	−2.41	5.30	0.0015*	−1.05	2.07	0.0438*
IL-8	−0.12	1.09	0.8465	−0.71	1.64	0.2333
IL-10	1.41	0.38	< 0.0001*	−1.81	3.50	<0.0001*
IL-17A	−1.58	2.99	0.0035*	−0.13	1.10	0.281
TGF-β	0.31	0.8	0.4876	−0.63	1.55	0.3598

Statistical test: unpaired t-test, α = 0.05. ΔΔC_T_ = delta delta C_T_, FC = fold change, * = significant at α = 0.05.

Except for IL-1β and IL-10, the mRNA expression levels for most of these cytokines showed similar pattern both in blood and biopsy samples. Unlike in blood samples, the mRNA gene expression for IL-1β was significantly higher in the skin biopsies of untreated ENL patients than in LL patient controls. On the other hand, the gene mRNA expression for IL-10 was significantly lower in the biopsies of untreated ENL than in LL controls (Table 2).

### The Kinetics of Cytokines Gene Expression in the Blood Samples within ENL Patient Group before and after Treatment

The gene expression of the cytokines was also investigated in blood samples from patients within ENL group before and after treatment. The ΔΔC_T_ of each pair was obtained by subtracting the ΔΔC_T_ before treatment from ΔΔC_T_ after treatment (ΔΔC_T__after_ − ΔΔC_T__before_).

The mRNA expression for IL-8 and TGF-β did not show a statistically significant difference before and after treatment. On the other hand, the gene expression levels of IFN-γ (*P* < 0.0001), IL-6 (*P* ≤ 0.0001), IL-1β (*P* ≤ 0.005), and TNF (*P* ≤ 0.05) were significantly decreased in the blood samples from patients with ENL after treatment while it was significantly increased for IL-10 (*P* ≤ 0.0001) (Table 3).

**Table 3:** Cytokine mRNA expression in the blood samples from erythema nodosum leprosum before and after treatment.

Cytokine	ΔΔC_T_	FC	*P*-value	Gene expression after treatment
TNF	1.19	0.44	0.0497*	Decreased
IFN-γ	1.61	0.33	<0.0001*	Decreased
IL-1β	1.56	0.34	0.0043*	Decreased
IL-6	1.60	0.33	<0.0001*	Decreased
IL-8	0.48	0.72	0.0790	No change
IL-10	−1.32	2.50	<0.0001*	Increased
IL-17A	2.34	0.20	<0.0001*	Decreased
TGF-β	0.24	0.85	0.7160	No change

Statistical test: paired t-test, α = 0.05. ΔΔC_T_ = delta delta C_T_; FC = fold change; * = significant at α = 0.05.

### The Kinetics of Cytokines Gene Expression in the Skin Biopsy Samples within ENL Patient Group before and after Treatment

The mRNA expression in biopsy samples for TNF (*P* ≤ 0.0001), IFN-γ (*P* ≤ 0.0001), IL-1β (*P* ≤ 0.0005), and IL-17A (*P* ≤ 0.0204) were significantly decreased after prednisolone treatment. On the other hand, mRNA expression level for IL-10 was considerably increased after treatment (*P* ≤ 0.0001). The mRNA expression for IL-6, IL-8, and TGF-β did not show statistically a significant difference before and after treatment (Table 4).

**Table 4:** Cytokine mRNA expression in skin biopsy samples from patients with erythema nodosum leprosum before and after treatment.

Cytokine	ΔΔC_T_	FC	*P*-value	Gene expression after treatment
TNF	2.34	0.2	<0.0001*	Decreased
IFN-γ	3.03	0.12	<0.0001*	Decreased
IL-1β	2.39	0.19	0.0005*	Decreased
IL-6	0.31	0.81	0.6451	No change
IL-8	0.16	0.90	0.7617	No change
IL-10	−1.51	2.85	<0.0001*	Increased
IL-17A	1.01	0.5	0.0204*	Decreased
TGF-β	0.09	0.94	0.8337	No change

Statistical test: paired t-test, α = 0.05. ΔΔC_T_ = delta delta C_T_, FC = fold change, * = significant at α = 0.05.

## Discussion

The *in vitro* cytokine response of PBMCs from patients with ENL and LL controls to *M. leprae* antigen and its gene expression in blood and skin biopsy samples were compared before, during, and after treatment. Some of the key findings are discussed in the following paragraphs.

The analysis of ROC curve has shown that none of these cytokines are good enough to discriminate ENL from non-reactional LL patient controls. This indicates that multiple cytokines are involved in the pathogenesis of ENL. Among the studied cytokines TNF, IL-6, and IL-7A seem more powerful to discriminate ENL from LL although the discriminating power for these cytokines is less than 80%.

### TNF

The *in vitro* response of TNF to *M. leprae whole-cell sonicates* (MLWCS) in the PBMCs from patients with ENL and LL controls was investigated before and after treatment. The mean production of TNF in response to *M. leprae* antigen stimulation was significantly higher (83.6.4 ± 18.8, SE pg/mL) in the culture supernatants of PBMCs from patients with ENL than from LL patient controls (19.4 pg/mL ± 10.44) before treatment. However, TNF production was not significantly different in these groups after treatment. Similar findings have been reported by several studies (5, 37, 38). On the other hand, the detection of TNF in plasma or serum samples from patients with ENL has not been consistently reported. Some authors did not find a significant difference in patients with ENL and LL controls (39, 40). Others reported that increased TNF production occurred during corticosteroid treatment and decreased after treatment (9, 41–43). Interestingly, an upregulation of TNF production after thalidomide treatment of patients with ENL was also reported (41). These authors justified the increasing production of TNF during thalidomide treatment as an indication of immune stimulation. However, thalidomide and prednisolone may have different effect on the TNF. The variation of the results in various studies can be attributed to several factors such as experimental design, sample size, ENL definition, assay sensitivity, and assay methods.

In our study, the mRNA gene expression for TNF in blood and skin biopsies from patients with ENL was significantly upregulated compared to in LL controls before treatment. After treatment, the level of gene expression for TNF in blood and skin biopsy samples from patients with ENL was significantly decreased. Similar findings have been reported by previous studies (5, 21, 22, 38). On the other hand, the absence of any significant difference regarding the gene expression of TNF in the skin biopsies from patients with ENL and LL controls was also reported by Yamamura et al. (6).

Thus, our present kinetic study confirmed that TNF is certainly increased during ENL reactions in the same individual both *in vitro* (following stimulation of the cells with MLWCS) and *in vivo* (increased TNF gene expression *in situ*). TNF is a cell signaling cytokine involved in systemic inflammation and it is one of cytokines that contribute to the acute phase reaction (27). However, excessive production of TNF can cause tissue injury. Hence, our data imply that TNF is involved in the pathogenesis of ENL and it may be of use for the diagnosis of ENL. Investigating the sources of TNF (identifying the major immune cells producing TNF) in the pathogeneses of ENL may also be important to explore alternative therapeutics in the future.

### IFN-γ

In the present study, the mean production of IFN-γ in response to *M. leprae* antigen stimulation was significantly higher (1361 pg/mL ± 309.6) in the culture supernatants of PBMCs from patients with ENL than from LL patient controls (280.1 pg/mL ± 269.8) before treatment (*P* ≤ 0.05). However, IFN-γ production was not significantly different in both groups after treatment of ENL patients. Previous independent studies have also reported an increasing *in vitro* production of IFN-γ in untreated patients with ENL to the response of *M. leprae* stimulation (7, 19, 44). IFN-γ mRNA gene expression in the blood and skin biopsies from patients with ENL was significantly upregulated compared to in LL patient controls before prednisolone treatment. After treatment, the level of IFN-γ gene expression in the blood and skin biopsy samples from patients with ENL was significantly decreased. Similar findings have previously been reported by Iyer et al. (7) and Moraes et al. (20).

It should be noted that although fewer investigations have focused thus far on the role of IFN-γ, in contrast to the weight given to TNF, the results are more consistent and indicate an important role of IFN-γ in the immunopathology and occurrence of ENL. One earlier study has reported that administration of recombinant IFN-γ to patients with LL led to the development of ENL reactions in 60% of the patients over a 6- to 7-month period compared with an incidence of 15% per year with multiple drug therapy alone (45). Previous studies have demonstrated that monocyte/macrophage TNF-α production can be enhanced by the synergistic effect of IFN-γ (46). In addition, IFN-γ priming of peripheral blood monocytes from patients with leprosy has been demonstrated to enhance TNF-α production, both *in vivo* and *in vitro* (45). The mechanism by which IFN-γ induces ENL reactions in patients with leprosy is suggested to be through priming of monocytes, resulting in enhanced TNF production (47). IFN-γ is a major macrophage activator and a known inducer of macrophage TNF. It increases antigen presentation and lysosome activity of macrophages. However, over expression of INF-γ has been associated with the pathogenesis of a number of inflammatory and autoimmune diseases (48). The finding of increased INF-γ gene expression and production in untreated ENL patients in our study underlines the strong association between INF-γ and ENL reaction. Hence, IFN-γ is another candidate cytokine which may help to search for alternative effective drug for ENL treatment.

### IL-6

In the present study, we found that the *in vitro* response of IL-6 production was substantially higher in patients with ENL than in LL controls before treatment. IL-6 mRNA gene expression in the blood and skin biopsy samples was also significantly upregulated in patients with ENL than in LL controls. The *in vitro* production and gene expression of this cytokine was decreased after prednisolone treatment of patients with ENL and a significant difference was not observed in the two groups after treatment. However, its gene expression in the skin samples was not appreciably decreased in patients with ENL after prednisolone treatment. This suggests that although the systemic symptoms of ENL subside after treatment, there could be an ongoing immune response locally in the skin lesions which could take a longer time to establish immune homeostasis. Similar findings have been reported by several authors (6, 20, 30, 49). IL-6 is an interleukin that acts as both a pro-inflammatory and an anti-inflammatory cytokine which is secreted by T cells and macrophages (50). It is an important cytokine-mediating fever and the acute phase response through its ability of crossing the blood–brain barrier and initiating the synthesis of prostaglandin E2 in the hypothalamus thereby changing the body’s temperature set point (51). Hence, it is sensible to assume that higher levels of IL-6 production could contribute to the development of ENL reactions in non-reactional LL patients mainly owing to the potent pro-inflammatory role of IL-6 and its capacity to stimulate antibody production. The increased production of IL-6 in ENL reactions due to polymorphisms in the genes encoding the cytokine has been suggested (52). Based on the multiple effects of IL-6 on the control of innate and adaptive immune responses, its potential contribution to the immunopathogenesis of ENL reaction needs to be explored further.

### IL-8

In this study, higher *in vitro* production of IL-8 to the response of PBMCs stimulation to *M. leprae* was obtained in patients with ENL than in LL controls before, during, and after prednisolone treatment. Unlike the other cytokines studied, IL-8 production did not decrease during and after prednisolone treatment of patients with ENL. The mRNA gene expression of IL-8 both in the blood and skin biopsy samples was also significantly upregulated in patients with ENL before, during, and after prednisolone treatment. Few studies have investigated the role IL-8 from serum in the immunopathogenesis of ENL by comparing its production and gene expression. IL-8 mRNA upregulation in untreated patients with ENL has been previously reported by some authors (6) and its *in vitro* production (31) and both studies are in agreement with our finding.

IL-8 (CXCL8) is a chemokine produced mainly by macrophages and plays a key role in inflammation in neutrophil recruitment and inducer of phagocytosis. Studies have shown that anti-IL-8 treatment prevent neutrophil-dependent tissue damage as well as neutrophil infiltration in several types of acute inflammatory reactions, including lipopolysaccharide (LPS)-induced dermatitis, LPS/IL-1-induced arthritis and acute immune complex-type glomerulonephritis (53).

Thus, the finding of increased IL-8 production as well as IL-8 mRNA gene expression in the blood and skin lesions from patients with ENL suggests a potential role for this chemokine in the pathogenesis of ENL. Hence, exploring the potential role of IL-8 in the immunopathogenesis of ENL may provide valuable information in the diagnosis and treatment of ENL.

### IL-17

The *in vitro* production of IL-17A in PBMC samples and IL-17 mRNA gene expression in the blood and skin biopsy samples from patients with ENL was significantly increased before treatment and decreased after prednisolone treatment. IL-17A is the least studied cytokine in ENL reaction. Recently a cross-sectional study has reported that increased IL-17A production to *M. leprae* stimulation in ENL patients compared to non-reactional LL patients (26). One study has reported the upregulation of IL-17A before and after thalidomide treatment of ENL patients (24). However, the effect of thalidomide on IL-17A expression may be different from that of prednisolone. IL-17A is an immunoregulatory cytokine capable of promoting the generation of pro-inflammatory cytokines and chemokines, which leads to the attraction of neutrophils and macrophages to the inflammation site (54). The finding of increased IL-17A production and its mRNA gene expression in patients with ENL in the present study shows the involvement of IL-17A in the pathogenesis of ENL reaction. Hence, understanding the exact role of this cytokine in ENL reaction will benefit the development of novel immune modulators that reduce inflammation and thereby protect tissue damage in patients with ENL.

### IL-10

The *in vitro* production of IL-10 in response to *M. leprae* stimulation in the PBMCs of patients with ENL was significantly lower than that in LL controls before treatment. After prednisolone treatment of ENL patients, IL-10 production was significantly increased and it was higher than the value obtained in LL controls. The present result is in agreement with Sampaio et al. (38). Although IL-10 mRNA gene expression in the blood samples before and after treatment in patients with ENL and LL controls did not reveal a statistically significantly different result, the longitudinal comparison in patients with ENL has shown significantly increased IL-10 mRNA gene expression after prednisolone treatment similar to the *in vitro* IL-10 production. On the other hand, unlike the blood samples, the gene expression in skin biopsy samples was significantly decreased before treatment in patients with ENL compared to LL controls and considerably increased after prednisolone treatment. Therefore, it seems that IL-10 regulates excess immune response locally in the skin than systemically in the blood. Variable and inconstant reports have been published on serum or plasma IL-10 production in patients with ENL. Increased IL-10 production in patients with ENL has been reported by some authors (9, 40). Other studies failed to detect any differences in serum IL-10 between patients with ENL and LL controls (7, 25, 43) or in IL-10 mRNA in biopsy skin specimens (22).

IL-10 is a well-known cytokine involved in downregulating macrophage functions. IL-10 has been shown to inhibit cytokine synthesis by monocytes, namely TNF-α, IL-1, IL-6, IL-8, and IL-12 (38). Thus, the finding of decreased IL-10 production as well as IL-10 gene expression in untreated patients with ENL reaction implies the loss of control over these pro-inflammatory cytokines which exacerbates ENL reactions.

In conclusion, the *in vitro* production and gene expression of the cytokines: TNF, IFN-γ, IL-6, IL-8, and IL-17A were significantly increased in untreated patients with ENL at recruitment. However, IL-10 production and gene expression was significantly lower in untreated ENL patients and significantly increased after prednisolone treatment. This is the first study examining the effect of prednisolone on the kinetics of inflammatory cytokines in patients with ENL reactions before and after treatment. Our findings suggest that prednisolone modulates the pro-inflammatory cytokines studied here either directly or through suppressing the immune cells producing these inflammatory cytokines. This needs further confirmation through identification of the immune cells producing these cytokines. Prednisolone is extensively used for treatment of ENL reactions, but clinical improvement varies, and a better understanding of the immunology of ENL is required to improve treatment for these patients. Although the immune response to *M. leprae* is cytokine mediated, the involvement of cytokines in ENL reactions is less understood. Our data clearly suggest that cytokines are involved in ENL reaction, yet the sources of these cytokines are unknown. If prednisolone effectively switch off these inflammatory cytokines, why some ENL patients do not show clinical improvement to prednisolone treatment? Therefore, cytokines may not be the only key players in the pathogenesis of ENL. There could be other factors or modulators other than cytokines which take part in the immunopathogenesis of ENL. These assumptions need to be further investigated and we need to understand how prednisolone works with cytokines which could lead us to better drugs that effectively resolve inflammation more rapidly.

## Ethics Statement

Informed written consent for blood samples were obtained from patients following approval of the study by the Institutional Ethical Committee of London School of Hygiene and Tropical Medicine, UK (#6391), AHRI/ALERT, Ethiopia (P032/12) and the National Research Ethics Review Committee, Ethiopia (#310/450/06). All data have been analyzed and reported anonymously.

## Author Contributions

EN and DL formulated the study questions. EN, DL, HD, SW, MA, and KB designed the study protocol. EN, BE, AT, YB, and KB conducted the experiment. AA, HD, and DL supervised the study. EN analyzed the data. All authors contributed to the interpretation of the data. EN drafted the manuscript. KB, SW, BE, MA, AT, YB, AA, HD, and DL revised the manuscript. All authors read and approved the final version for publication. All authors agreed to be accountable for all aspects of the work in ensuring that questions related to the accuracy or integrity of any part of the work are appropriately investigated and resolved.

## Conflict of Interest Statement

The authors declare that the research was conducted in the absence of any commercial or financial relationships that could be construed as a potential conflict of interest.
